# The Ross Procedure: Historical Context, Modern Outcomes, and the Road Ahead

**DOI:** 10.1007/s11886-025-02296-7

**Published:** 2025-10-23

**Authors:** Christian Eidson, Andrew Well, Joseph Turek, Carlos M. Mery, Ziv Beckerman

**Affiliations:** 1 Vanderbilt University School of Medicine, Nashville, TN USA; 2 Department of Cardiac Surgery, Division of Pediatric Cardiac Surgery, Vanderbilt University School of Medicine, Nashville, TN USA; 3 Pediatric Heart Institute, Monroe Carrel Jr. Children’s Hospital at Vanderbilt, Nashville, TN USA; 4 Division of Cardiovascular and Thoracic Surgery, Department of Surgery, Duke University Medical Center, Durham, NC USA; 5 2200 Children’s Way Suite 5143, Nashville, TN 37232 USA

**Keywords:** Ross Procedure, Aortic Valve Replacement, Aortic Valve Disease, Outcomes

## Abstract

**Purpose of Review:**

There remains no single perfect aortic valve replacement option in patients with aortic valve disease. In this manuscript, we will discuss the modern role of the Ross Procedure in children and adults with aortic valve disease.

**Recent Findings:**

Currently, the Ross procedure is the only established operation that allows replacement of the aortic valve with living, growing tissue. Use of the Ross procedure for aortic valve replacement has waxed and waned in recent decades. However, in recent years, with reports from long-term data series, interest has been redirected at the Ross procedure due to its excellent durability and freedom from long-term valvular morbidity and mortality.

**Summary:**

In patients requiring aortic valve replacement, the Ross procedure offers excellent short- and long-term outcomes and can be tailored to specific pathologies and the needs of individual patients. In this patient population, it is the only intervention that restores life expectancy to that of the general population. The Ross procedure remains a superb intervention for the treatment of aortic valve disease in children and adults in the modern era and should be heavily considered as an option when aortic valve replacement is required.

## Introduction

Diseases of the aortic valve, congenital and acquired, result in significant morbidity and mortality, with aortic stenosis (AS) carrying significant mortality risk, particularly if left untreated [1]. In the United States, the incidence of severe AS has remained stable, but the absolute burden of disease has increased due to growth of the population [2]. Aortic insufficiency (AI) also carries a severe burden, with mortality 2 years after diagnosis reaching up to 20.7% for patients with severe AI without inervention [3]. 

Surgical options for addressing aortic valve disease include aortic valve repair, surgical aortic valve replacement (SAVR) (mechanical, bioprosthetic valve, or homograft root), transcatheter aortic valve replacement (TAVR), the Ross procedure, and most recently, living-aortic root replacement. Each option has specific risks and benefits, and selection of the appropriate intervention can be complex.

The Ross procedure was first described in 1967 by Donald Ross, who employed a patient’s native pulmonary valve as a living autograft to replace a stenotic aortic valve. His aim was to use the transplanted pulmonary valve, as it is living, viable tissue, as a more durable autograft in the aortic location. The excised pulmonary valve was then replaced with a homograft, which performed very well under the reduced pressures of the pulmonary circulation [4]. 

In recent years, the Ross procedure has gained a resurgence in attention as a treatment option for pathologies of the aortic valve in children and non-elderly adults. Perhaps the most notable advantage of the Ross procedure is that it is the only aortic valve replacement option that has been shown to result in similar patient life expectancy to that of the general population [5–9]. In the oldest Ross experience reported by Dr. Yacoub, which included 108 adults with a median age of 38 years, of which 8% had active endocarditis and 42% were undergoing reoperations, there was 1(0.9%) perioperative death and twenty-five-year survival was 83.0% (95%CI: 75.5%−91.2%). This represented a relative survival of 99.1% (95%CI: 91.8%−100%) compared with the general population. Additionally at 25 years, freedom from any reintervention was 71.1% (95%CI: 61.6%−82.0%); with freedom from autograft reintervention of 80.3% (95%CI: 71.9%−89.6%); and from homograft reintervention of 86.3% (95%CI: 79.0%−94.3%). Thirty-day mortality after the first Ross-related reintervention was 0% and after all Ross-related reinterventions was 3.8% (*n* = 1). Ten-year survival after reoperation was 96.2% (95%CI: 89.0%−100%) [8]. 

Due to the renewed interest, this manuscript will review the modern role of the Ross procedure for treatment of aortic valve disease in children and adults, with a focus on procedural development and variations, patient selection, the role of the Ross procedure in patients with connective tissue disorders, and future advancements such as Partial Heart Transplantation (PHT) and the Living Ross Procedure.

## Intra- and Perioperative Considerations

### Standard Procedure Overview

**Fig. 1:**
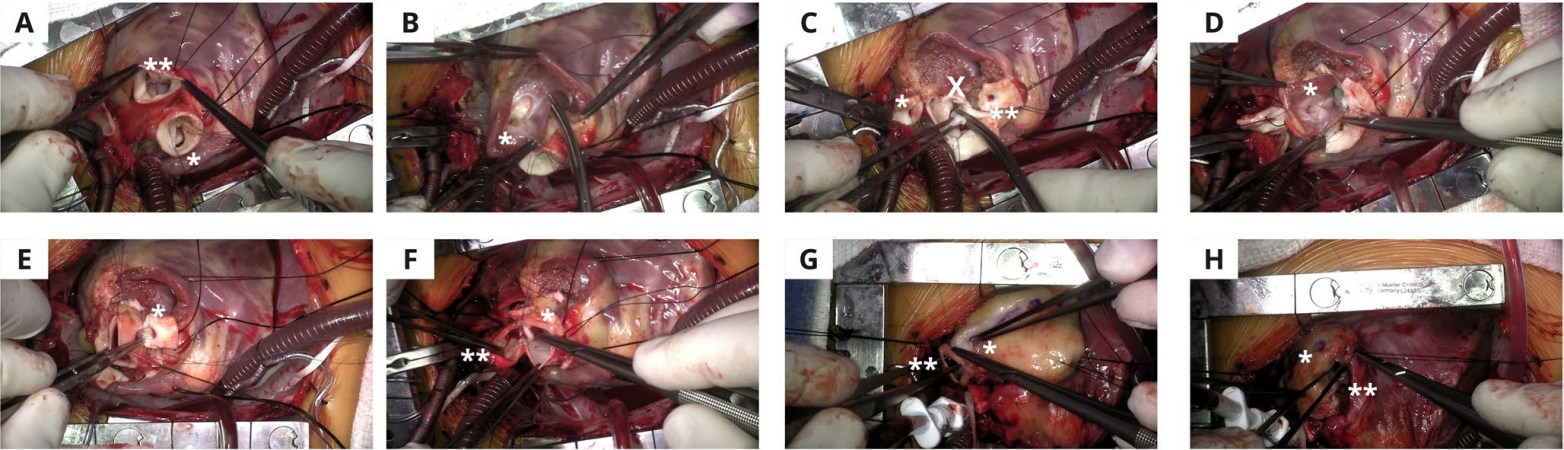
Key Steps for an Unreinforced Ross Procedure in a Young Child **A**: After a Sternotomy and initiation of cardiopulmonary bypass with aortic and bicaval cannulation the Aorta is transected and the Aortic valve(*) is inspected. If repair is deemed impossible, the Main Pulmonary Artery is transected and the Pulmonary Valve(**) is inspected **B**: If the Pulmonary Valve is deemed good to use as an autograft, the Pulmonary Valve(*) is meticulously excised to ensure no damage to surrounding structures occurs **C**: After excision of the Pulmonary Valve, the left (*) and right (**) coronary buttons are created and the native Aortic Valve leaflets (X) are excised **D**: The autograft (*) is then sutured into the left ventricular outflow tract **E**: The coronary buttons (*) are anastomosed to the autograft **F**: The anastomosis between the autograft (*) and the ascending aorta (**) is performed **G**: In this case, a homograft (*) is utilized for the Right Ventricle to Pulmonary Artery conduit and the anastomosis between the homograft and the distal Main Pulmonary Artery (**) is performed **H: **The proximal end of the homograft (*) is then sutured into the Right Ventricular Outflow Tract (**)

The Ross procedure includes the completion of the following steps: excision of the aortic valve, en-bloc harvesting of the pulmonary valve and root, implantation of the harvested pulmonary valve in the aortic position, implantation of the coronary buttons, reconstruction of aortic continuity, and placement of a right ventricle to pulmonary artery (RV-PA) conduit. (Fig. 1)

Briefly, after a median sternotomy and cannulation for standard cardiopulmonary bypass, aortic cross clamp is applied, and the heart is protected with cardioplegia. The aorta is then transected above the sinotubular junction, and the aortic valve is inspected. If the decision is made to proceed with aortic valve replacement (i.e. repair of the aortic valve is not feasible), then the aortic valve leaflets are excised.

Next, the pulmonary artery is transected at the level of the takeoff of the right pulmonary artery and the pulmonary valve is inspected. If deemed adequate, the autograft is carefully harvested, with careful attention to avoid damage to the valve or surrounding structures. Once the autograft is explanted, it is preserved in a cold saline solution (4–10℃). Attention is then redirected to the aortic root. The aortic root complex is dissected out and the coronary artery buttons are mobilized.

The implantation of the autograft then follows and must be tailored to each patient’s anatomic features. The implantation should be at/below the native aortic valve annulus. It is critical that the pulmonary valve cusps are not distorted, and the symmetry of the valve is preserved during implantation.

Next, the coronary buttons are re-implanted in their respective sinuses. The autograft is then trimmed at the level of the aortic commissures, and aortic continuity is re-established while inserting an interposition graft (to stabilize the new sinotubular junction, in patients that don’t require future growth of the autograft). The implantation of the RV-PA conduit can either be done under aortic cross-clamp, or with the heart beating (surgeon’s preference). The conduit is most frequently a cryopreserved homograft, though either a composite of a graft-bioprosthesis, or most recently, a “living-root” RV-PA conduit can be used. Regardless of the chosen implant, the implantation should focus on achieving patent distal and proximal connections without distortion or damage to surrounding structures.

As the need for cardiac reinterventions after a Ross most frequently involves the RV-PA conduit, every effort should be made to plan ahead and design the operation in a way that would preferably allow safe transcatheter interventions down the road. Coronary compression during catheter balloon inflation, is the most common cause of inability to place a transcatheter valve after a Ross operation. This can be the result of : (1) Dilatation of the autograft, resulting in displacement of the coronary arteries, (2) Initial positioning of the coronary buttons in a non-anatomical location- usually with the left main coronary rotated clockwise in the aortic root and closer to the RV-PA conduit. Similar limitations can also be caused by the right coronary, but less frequently. Surgical decision making during the Ross operation should keep that in mind, to further optimize the overall outcome of Ross patients.

## Procedural Variation: the Reinforced Ross Procedure

Concerns surrounding the Ross operation are typically divided into 2 areas: the autograft, and the RV-PA conduit. Accordingly, the surgical technique has been adjusted over the years to further optimize and address these concerns.

For the autograft, the most common concern relates to dilation over time, potentially leading to aortic insufficiency. As such, various options for autograft reinforcement have been developed as modification to the standard Ross procedure. Reinforcement options can be directed to: (1) The sinotubular junction, (2) The left ventricular outflow tract (LVOT), (3) The entire autograft- by placing the entire autograft within a Dacron tube for reinforcement [10–15]. These adjustments have led to a marked improvement in outcomes according to different case series [12, 16, 17]. 

A 2020 study reported that patients who underwent the reinforced Ross procedure had a lower incidence of neoaortic root dilation at intermediate follow-up compared to patients who underwent the standard Ross procedure [16]. Similarly, a 2015 study found that at 1 and 3-year follow-up, patients who underwent the reinforced Ross technique were less likely to require reintervention or have neoaortic root dilatation. Furthermore, freedom from progressive neoaortic dilatation for the standard Ross cohort at the 6-month, 1-year, and 3-year follow-up (78%, 65%, and 52%, respectively) was consistently below that of the reinforced Ross cohort (90% consistently) [18] (Table 1).

**Table 1:** Ross Procedure Surgical Options

Autograft	Right Ventricle – Pulmonary Artery Conduit
Traditional Unreinforced	Preserved Homograft
Reinforced Sinotubular Junction Left Ventricular Outflow Tract Entire Autograft	Bovine Jugular Vein Graft
	Porcine Bioprosthesis
	Handmade ePTFE
	Living Homograft

## Procedural Variation: the RV-PA Conduit

Options for the RV-PA conduit include pulmonary homograft, bovine jugular vein (BJV) conduits, xenografts, expanded polytetrafluoroethylene (ePTFE) conduits, and tailor-made conduits. Concerns include conduit degeneration resulting in stenosis and/or insufficiency, endocarditis, and resultant need for reintervention.

Pulmonary homografts are the most commonly used conduits, with favorable hemodynamic properties and biocompatibility, but they have potentially limited availability particularly at smaller sizes [19]. Bovine jugular vein conduits are commonly used and have a lower progression of mean RV-PA conduit gradient compared to homografts, but higher rates of endocarditis, particularly late endocarditis [19–21]. Xenografts include stentless porcine bioprostheses, can provide an alternative to homografts [22]. Conduits using ePTFE are novel and handmade, and show some encouraging mid-term results with similar data to pulmonary homografts [23, 24]. Tailor-made conduits can incorporate autologous pericardium and ePTFE patches to create a custom RV-PA conduit and have demonstrated durable long-term function [25]. 

The progression of stenosis of the RV-PA conduit varies by the type of conduit, with BJV conduits demonstrating the lowest progression rate (1.45mmHg/year) compared to homografts (2.6 mmHg/year) and xenografts (2.9 mmHg/year). While insufficiency of the RV-PA conduit can result from deterioration it is less frequent than stenosis with a freedom from insufficiency (grade ≥ 2) of 95% after 14 years [19]. 

A critical measure of conduit durability is rate of reintervention. The freedom from RV-PA conduit intervention or dysfunction was found to be 90.6% and 79.5% at 15 years respectively [19, 26]. Although concerns over the RV-PA conduit have been a limiting factor for more widespread use of the Ross, new advancements (see “Future” section) in the field are hopeful to address this issue at a larger scale.

While data from adults undergoing RV-PA conduit placement, as above, allows for comparison of performance between conduit types over time, the pediatric population adds the additional factors of conduit size placed and its interaction with a patient’s growth. In one study of pediatric RV-PA conduits, freedom from conduit reintervention for all conduits was 68.8% at 10 years, 54.1% at 20 years, and 47.4% at 28 years [27]. 

## Patient Selection

A foundation in the success of the Ross procedure is thoughtful patient selection. In infants (< 1 year of age), the Ross procedure carries a higher risk of perioperative and short-term morbidity and mortality compared to patients > 1 year of age. However, this is associated with the increased underlying anatomical complexity of patients requiring Ross at < 1 year of age [28]. Specifically, a Ross procedure at an age < 84 days appears to be the point at which the risk for mortality and LVOT reintervention increases [29]. However, compared to other forms of SAVR, the Ross carries a lower incidence of late mortality (0.76% per year) and provides a 10-year overall survival rate of 78.9%.^30^ Additionally, the autograft maintains superior durability, with a freedom from LVOT reintervention up to 93–96% at 15 years [30]. While current options for aortic valve replacement in this population remain sub-optimal, the Ross procedure remains a promising option due its growth potential and long-term outcomes [6, 8]. 

For pediatric populations > 1 year of age, the Ross procedure has optimal outcomes for those with isolated aortic valvular disease, regardless of age. A 2023 study demonstrated 10-year overall survival following the Ross procedure to be 96.2% in children > 1 year of age, with a freedom from autograft reoperation of 86.0% at 10 years [30]. While reinforcement of the Ross is not typically utilized in the pediatric population to allow for continued growth, the Melbourne group reported their experience using an absorbable PDS band at the sinotubular junction which revealed 100% freedom from moderate or greater AI at 10 years compared to 83.1% in patients where the PDS band was not iutilized [28]. 

In adults, current guidelines recommend the Ross procedure for treating aortic valve disease in patients aged < 50 years who do not desire mechanical SAVR, are not already on anticoagulation, and who have appropriate anatomy and tissue characteristics [31]. In adults aged 50–65 years, the Ross procedure may considered for patients who have an anticipated life expectancy of at least 15 years, suitable anatomy, no other major cardiac comorbidities, and an active lifestyle [32]. Young adults fare exceedingly well, with studies showing that freedom from any re-operations after the Ross procedure was 89% after 10 years and 83% after 15 years. Furthermore, freedom from redo autograft surgery was 90% after 10 years and 81% after 15 years, and freedom from redo RV-PA conduit surgery was 89% after 10 years and 78% after 15 years. Overall, these data demonstrate that the Ross procedure maintains favorable long-term outcomes and freedom from re-intervention [32, 33]. 

For patients with congenital bicuspid aortic valves, the Ross operation offers excellent outcomes with late survival at 10 and 20 years post-Ross of 99% and 95%, respectively and freedom from autograft reoperation and/or more-than-mild AI at 10 and 20 years were 89% and 85%, respectively [34]. 

The Ross procedure has an inherent advantage in patients contemplating pregnancy. Women with mechanical aortic valves have higher risks of cardiac, thrombotic, and obstetric adverse events [31, 35]. Additionally, the necessary anticoagulation is teratogenic and complicates pregnancy [36, 37]. 

Overall, when carried out in skilled surgical centers with adequate operative volumes, the Ross procedure is associated with superior long-term outcomes when compared to prosthetic AVR. In children and non-elderly adults undergoing AVR, the Ross procedure is currently the only operation which restores long-term survival rates that are equal to those of the age-matched healthy general population [6, 9, 11, 32]. 

## Short-Term Considerations

### Perioperative Mortality

The Ross procedure has been found to have a perioperative mortality ranging from 0.3 to 1.1% depending on the series [17, 38–41]. This compares favorably to isolated SAVR which has perioperative mortality rates from 1.1 to 2.6% and TAVR perioperative mortality rates from 0.8–5.2%. [6, 42–44] These comparisons should be viewed with the understanding that there are differences in the characteristics of patients undergoing each intervention.

## Long-Term Outcomes

### Mortality

As described before, the Ross procedure is associated with excellent long-term outcomes and provides patients the potential of matching the life expectancy of the general population, which has not been demonstrated in any other aortic valve replacement option [9]. These findings have been reproduced with recent data demonstrating Ross procedure patients having superior unadjusted survival at 20 years compared to mechanical SAVR (95% vs. 68%; *p* < 0.001) and superior survival over the same period in a propensity-score matched analysis (94% vs. 84%, *p* = 0.018).^38^ Similar results of superior long-term survival for the Ross procedure compared to a matched bioprosthetic SAVR population have been found [9, 45]. While adequate primary valve repair remains the preferred intervention, a 2022 study of isolated congenital AS in children revealed mortality at 10 years of 0.9% in the primary repair group compared to 1% at 13 years in the Ross procedure [46, 47]. 

### Reintervention Rates

An important point of comparison for patients undergoing aortic valve interventions is the rate of reintervention. This is of specific concern in the Ross procedure with the potential need for reintervention on the RV-PA conduit in addition to the autograft. However, series in older children and adults have demonstrated a freedom of autograft reintervention of > 80% at 10 or more years and an overall freedom from reintervention rate of greater than 70%. [6, 9, 48] While freedom from reintervention is high after the Ross procedure, it still lags recent reports of 10-year freedom from reintervention in all-comer mechanical SAVR of 95.3% and bioprosthetic SAVR of 94.2%. [49] This difference remained when evaluating only those < 60 years of age with 10-year freedom from reintervention of 95.2% for mechanical SAVR and 87.7% in bioprosthetic SAVR [49]. 

Evaluation of the long-term risks of primary repair of the aortic valve is challenging due to differences in the underlying anatomy and type of repair done, but in general primary repair has freedom from reintervention rates that range from 8.5 to 67.9% at 10 years [46, 50, 51]. 

With regards to long-term aortic valve function, the Ross procedure has higher rates of freedom from valvular complications such as AS and AI when compared to mechanical AVR [40, 45, 47]. 

### Additional Long-Term Risks

The Ross procedure has consistently been found to have significantly reduced long-term incidence of stroke (2.1% in the Ross vs. 4.8% following mechanical SAVR at 15 years post-operation) and major bleeding (1.9% following the Ross vs. 5.2% following mechanical SAVR) when compared to mechanical SAVR [9, 40, 52]. Compared to bioprosthetic valves specifically, the Ross procedure is associated with lower rates of endocarditis (2.3% following the Ross vs. 8.5% following bioprosthetic SAVR), thromboembolic events (0% following the Ross vs. 14.1% following bioprosthetic SAVR), and permanent pacemaker implantation (1.9% following the Ross vs. 15.9% following bioprosthetic SAVR) [9, 45]. These comparisons are similar for the timepoints at 5, 10, and 20 years [40]. 

### Special Populations: Role in Marfan’s Syndrome and Other Connective Tissue Disorders

Connective tissue disorders, such as Marfan’s syndrome, predispose patients to aortic root dilation and dissection [53–55]. Therefore, for some time now, it has been contemplated that the Ross operation in this setting may predispose the autograft to the same risks. However, with the development of the reinforced Ross operation, this risk may be addressable.

Data in patients with connective tissue disorder is limited to case reports. In one case, a modified Ross procedure was performed in a 47-year-old man with Marfan syndrome for moderate aortic insufficiency from a bicuspid aortic valve, mild mitral insufficiency, and a 4.8 cm dilated aortic root. The pulmonary autograft was sewn into a 32-mm polyester tube, and his aortic root was replaced with this autograft construct. The ascending aorta was replaced with a portion of this same graft material. At his most recent follow-up (8 years after the procedure), he demonstrated a stable aortic root with no aortic stenosis or insufficiency. The pulmonary valve that was replaced with allograft had no obstruction and mild insufficiency. He had no limitations on activity following the operation [56]. 

### Future: the Role of Partial Heart Transplant in the Living Ross

Recent exciting developments have been made that can further advance the utility and adaptability of the Ross in the modern era, potentially minimizing the potential need for RV-PA conduit reinterventions. The Partial Heart Transplant (PHT) or living root replacement includes implanting a “living root” tissue in the aortic and/or pulmonic positions as a full root replacement option. In this strategy, with providing short term immunosuppression, the conduits perform well (in short term studies) and demonstrate growth [57–59]. This has recently been performed by the authors of this manuscript in both pediatric and adult patients, as the first described “living-Ross” operations.

The graft is treated as an orthotopic heart transplant to maintain its cellular viability, which differentiates it from a homograft. The operative technique is similar to homograft valve replacement. This procedure allows the valve implants to grow, which may eliminate the need for reintervention, and increase long-term durability of the transplanted conduit [60]. 

PHT has a possibility to make the Ross procedure a truly curative intervention. The Ross is carried out in the same fashion as usual, with use of the patient’s native pulmonic valve as the autograft in the aortic position. Then, instead of a homograft in the pulmonic location, a transplanted living root from a donor is used; both valves could then grow with the recipient, theoretically decreasing the risk of RV-PA conduit dysfunction and thereby increasing long-term durability and freedom from reintervention of the Ross procedure.

The surgical risks of PHT are similar to those of conventional valve replacement operations in infants and young children, with the addition of immunosuppression. There is some evidence that heart valves carry some degree of immune privilege, which may lower the need for immunosuppression [61]. Furthermore, a fault in immunosuppression would likely render the partial heart transplant into a homograft, which is currently the standard intervention for the pulmonic location during a Ross procedure [60]. 

Clinical trials for PHT are currently ongoing, and the future of this procedure and its role in the treatment of irreparable heart valves are very promising, but are yet to be fully elucidated [60]. There are also studies that suggest there may be a role for tissue-engineered heart valves in the Ross procedure, which may further decrease valvular degeneration and improve hemodynamic performance (Huygens et al; Dohmen et al) [62, 63]. Incorporation of such techniques into the Ross operation can augment the existing excellent durability and long-term mortality benefits of the Ross procedure and further amplify its use in the modern era and in the future of AVR [64].

## Conclusions

In patients who are in need of aortic valve replacement, there is essentially no anatomic substrate that the Ross operation cannot be tailored to. The Ross operation is a sophisticated procedure, which requires experience and attention to details. The nuances of the operation allow the surgeon to tailor the operation to the patient, based on their unique anatomy, aortic root/LVOT dilatation, connective tissue characteristics, ascending aorta diameter, etc. In experienced hands, the immediate as well as the long-term outcomes are outstanding. Perhaps the only situation not amenable for a Ross operation would be in case of pulmonary valve pathology.

The fact that the Ross procedure is the only intervention that restores life expectancy to that of the general population cannot be understated. (Table 2) When performed in experienced centers with skilled surgeons and considerate patient selection, the Ross procedure provides excellent short and long-term outcomes. The Ross procedure remains an exceptional (and perhaps the best available) option for replacement of the aortic valve in children and adults in the modern era.

**Table 2:** Ross Outcomes

Series	Number of Patients	Mean/Median Age (Years)	Mean/Median Follow Up (Years)	Freedom from Reintervention	Perioperative Mortality	Survival
Bohm 2001^10^	186	38.2 [Range: 2–62]	2.3	NR	0%	NR
David 2014^5^	212	34 ± 9	13.8	All at 20 Years: 79.9% (95% CI: 61.7% − 87.9%)	0.5%	20 Years: 93.6% (95% CI: 88.1% − 96.6%)
Skillington 2015^17^	322	39.5 [Range: 15–63]	9.8	Autograft at 18 Years: 96% (95% CI: 92% − 98%)	0.3%	20 Years: 97%
Sharabiani 2016^6^	718	13.1 [IQR: 7.5–17.0]	6.6	Autograft at 12 Years: 91.1% (95% CI: 87.3% − 93.8%)	1.1%	12 Years: 97.3% (95% CI: 95.6% − 98.4%)
Sievers 2016^39^	1,779	44.7 ± 11.6	8.3	All at 15 Years: 82.7%	1.1%	
Poh 2018^34^	129	34.7 ± 10.6	9.6	Autograft at 20 Years: 85% (95% CI: 74% − 92%)	0.8%	20 Years: 95% (95% CI: 85% − 99%)
Pergola 2020^33^	536	29.4 ± 11.1	16.3	All at 20 Years: 75.3%	1.5%	NR
Aboud 2021^7^	2,444	44.1 ± 11.7	9.2	All at 25 Years: 61.5%	1.0%	25 Years: 75.8% (95% CI: 70.0% − 82.0%)
El-Hamamsy 2022^9^	434	35.9 ± 9.2	12.5	All at 15 Years: 82.8% (95% CI: 78.4% − 86.8%)	0.2%	15 Years: 93.2% (95% CI: 89.0% − 95.9%
Varrica 2022^15^	15	15 ± 1.4	15	NR	0%	NR
Cleveland 2023^30^	58	0.17 [Range: 0.02–0.43]	6.7	Autograft at 15 Years: 96%	19%	NR
Notenbloom 2024^8^	108	38 [Range: 19–66]	24.1	All at 25 Years: 71.1% (95% CI: 61.6% − 82.0%)	0.9%	25 Years: 83.0% (95% CI: 75.5% − 91.2%)
NR: Not Reported						

## Key References

- Notenboom ML, Melina G, Veen KM, et al. Long-Term Clinical and Echocardiographic Outcomes Following the Ross Procedure: A Post Hoc Analysis of a Randomized Clinical Trial. *JAMA Cardiol*. 2024;9(1):6-14. doi:10.1001/jamacardio.2023.4090
  - **Findings from this 2024 post-hoc analysis of a randomized clinical trial illustrate that the Ross procedure provides excellent survival into the third decade postoperatively (the longest post-operative follow-up to date), which is comparable to survival in the general population**.
- El-Hamamsy I, Toyoda N, Itagaki S, et al. Propensity-Matched Comparison of the Ross Procedure and Prosthetic Aortic Valve Replacement in Adults. *J Am Coll Cardiol*. 2022;79(8):805-815. doi:10.1016/j.jacc.2021.11.057
  - **Findings from this 2022 propensity-matched cohort study highlight that the Ross procedure is associated with superior long-term survival and freedom from valve-related complications compared with prosthetic (mechanical and bioprosthetic) surgical AVR.**
- Cleveland JD, Bansal N, Wells WJ, Wiggins LM, Kumar SR, Starnes VA. Ross procedure in neonates and infants: A valuable operation with defined limits. *J Thorac Cardiovasc Surg*. 2023;165(1):262-272.e3. doi:10.1016/j.jtcvs.2022.04.015
  - **Findings from this 2023 study highlight the utility of the Ross procedure in neonates and infants (as it offers excellent intermediate-term freedom from LVOT reintervention) as well as the importance of preoperative risk assessment and patient selection.**

## Data Availability

No datasets were generated or analysed during the current study.
